# New records of five polydesmidan millipedes (Diplopoda, Polydesmida) from the Ogasawara Islands, Japan, with notes on the postembryonic development of *Prosopodesmus* spp. (Haplodesmidae)

**DOI:** 10.3897/BDJ.14.e194219

**Published:** 2026-07-01

**Authors:** Soma Chiyoda, Yu Hisasue, Hiroki Yoshino, Ryosuke Kuwahara, Toru Miura

**Affiliations:** 1 Misaki Marine Biological Station, School of Science, The University of Tokyo, Koajiro Misakimachi, Miura, Kanagawa, 238-0225, Japan Misaki Marine Biological Station, School of Science, The University of Tokyo Koajiro Misakimachi, Miura, Kanagawa, 238-0225 Japan; 2 Ogasawara Division of Japan Wildlife Research Center, Okumura, Chichijima, Ogasawara, Tokyo, 100-2101, Japan Ogasawara Division of Japan Wildlife Research Center Okumura, Chichijima, Ogasawara, Tokyo, 100-2101 Japan; 3 Department of Biological Sciences, Tokyo Metropolitan University, Minami-Osawa, Hachioji, Tokyo, 192-0397, Japan Department of Biological Sciences, Tokyo Metropolitan University Minami-Osawa, Hachioji, Tokyo, 192-0397 Japan; 4 Graduate School of Integrated Sciences for Life, Hiroshima University, Kagamiyama, Higashihiroshima, Hiroshima, 739-8528, Japan Graduate School of Integrated Sciences for Life, Hiroshima University Kagamiyama, Higashihiroshima, Hiroshima, 739-8528 Japan

**Keywords:** alien species, anamorphosis, fauna, Myriapoda, oceanic islands, Opisotretidae, Pacific Ocean, Pyrgodesmidae

## Abstract

**Background:**

The Ogasawara Islands are remote oceanic islands located approximately 1,000 km south of mainland Japan and investigations of their fauna are essential for understanding the processes underlying the assembly of oceanic island biotas. Despite this importance, knowledge of several terrestrial invertebrate groups remains remarkably limited, including millipedes, which constitute a major component of soil macrofauna. Previous studies on the millipede fauna of the Ogasawara Islands have been sporadic and, to date, only ten identified species had been recorded from the archipelago.

**New information:**

Field surveys conducted between 2023 and 2026 on Ototo-jima, Ani-jima, Chichi-jima and Haha-jima Islands revealed five species of polydesmid millipedes: *Prosopodesmus jacobsoni* Silvestri, 1910; *P. panporus* Blower & Rundle, 1980 (Haplodesmidae); *Cryptocorypha ornata* (Attems, 1938); *Dyskolonius uniramus* (Attems, 1938) (Pyrgodesmidae); and *Solaenaulus butteli* (Carl, 1922) (Opisotretidae). All five species represent new records for both Japan and the Ogasawara Islands. Reexamination of specimens previously reported as *Prosopodesmus sinuatus* from the Ogasawara Islands revealed that they were misidentified and actually belong to *Cryptocorypha ornata*. Furthermore, the previous record of *Solaenaulus* sp. from Chichi-jima Island was identified as *S. butteli*, based on detailed sketches of the gonopods. In addition, we partially examined and compared the postembryonic development of *Prosopodesmus* species, a genus known for intrageneric polymorphism in the number of body rings in adult males. Based on these observations, we discuss potential heterochronic shifts in sexual maturation amongst species and between sexes.

## Introduction

The Ogasawara Islands are oceanic islands located approximately 1,000 km south of mainland Japan. Although their fauna is depauperate in terms of species richness as a result of geographic isolation, it nevertheless contains numerous endemic species and subspecies (Shimizu 2003, Karube 2004). Studies on fauna and flora of the Ogasawara Islands have suggested that their unique biota consists of multiple biogeographic elements: Palaearctic, Oriental (including Southeast Asian) and Micronesian (Takakuwa 2004). However, information on many terrestrial invertebrates, for example, small arthropods, remains limited and further accumulation of basic distributional data is needed to clarify the formation processes of the Ogasawara fauna (Hisasue and Tsuji 2024).

Furthermore, it should be noted that many plant and animal species have been intentionally or unintentionally introduced into the Ogasawara Islands through human activities. Amongst these, invasive predators, such as the green anole *Anolis carolinensis* Voigt, 1832, the cane toad *Rhinella marina* (Linnaeus, 1758) and the New Guinea flatworm *Platydemus manokwari* Beauchamp, 1962, have had severe impacts on the native terrestrial invertebrate fauna (Tomiyama 1998, Ohbayashi et al. 2007, Abe et al. 2008, Karube 2009). To assess these impacts and detect new introductions, regular monitoring of small terrestrial invertebrates, including soil animals, is essential.

Millipedes, as conspicuous components of the soil macrofauna, represent one of the groups that have long been overlooked in the Ogasawara Islands. Records of millipedes from the Ogasawara Islands were summarised by Yosioki Takakuwa in the 1940s (Takakuwa 1942a, Takakuwa 1943). Since then, although comprehensive surveys of the soil fauna have been conducted (Aoki and Harada 1978, Shimano et al. 2018), these studies did not explicitly examine the species composition of myriapods. Consequently, subsequent records of millipedes have remained sporadic, with the report by Tanabe (1991) being one of the few relatively comprehensive exceptions.

Millipede species recorded so far from the Ogasawara Islands are listed in Table 1. Ten species of millipedes, *Lophoturus okinawai* (Nguyen Duy-Jacquemin and Condé, 1982) (Polyxenida, Lophoproctidae), *Rhinotus okabei* (Takakuwa, 1942) (Polyzoniida, Siphonotidae), *Prosopodesmus sinuatus* (Miyosi, 1958) (Polydesmida, Haplodesmidae), *Helicorthomorpha holstii* (Pocock, 1895), *Nedyopus boninensis* Verhoeff, 1940, *Asiomorpha coarctata* (de Saussure, 1860), *Oxidus gracilis* (C. L. Koch, 1847) (Polydesmida, Paradoxosomatidae), *Anaulaciulus koreanus boninensis* (Verhoeff, 1939), *Japanioiulus lobatus* Verhoeff, 1937 (Julida, Julidae) and *Pseudospirobolellus avernus* (Butler, 1876) (Spirobolida, Pseudospirobolellidae), have been recorded from the Ogasawara Islands (Verhoeff 1939, Verhoeff 1940, Takakuwa 1940, Takakuwa 1942a, Takakuwa 1942b, Takakuwa 1943, Takashima 1950, Ishii 1988, Ishii 1997, Tanabe 1991, Shinohara 1993, Kuwahara and Hoshino 2025, Kuwahara et al. 2025). In addition, five unidentified species have been recorded (Takano 1988, Tanabe 1991, Nishimura and Takayama 2022). Although the records by Takakuwa 1942a and Takakuwa 1943 were published as part of a general distributional checklist without detailed collection data or taxonomic justification, we treated them as valid records and included them in Table 1. However, the record of *Symphyopleurium okazakii* (Takakuwa, 1942) from the Ogasawara Islands in Takakuwa (1943) is not treated as valid in this study. This is because it was not included in Takakuwa's later review (Takakuwa 1954) and its distribution is unlikely to extend to the oceanic Ogasawara Islands, given that previous records are restricted to inland areas of the Japanese main islands (e.g. Takakuwa 1942b, Minato 1969, Masuda 1973). Regarding the record of *A. koreanus boninensis* from the Ogasawara Islands, it has been suggested to result from a possible label mix-up, given that the same subspecies has been reported from geographically distant Korea (Paik 1958). However, unidentified species of the genus *Anaulaciulus* have subsequently been recorded from the islands (Tanabe 1991, Nishimura and Takayama 2022). Pending further verification, we therefore provisionally retain this record as valid.

To investigate the millipede fauna of the Ogasawara Islands and to discuss the processes underlying its formation, we conducted surveys on Ototo-jima, Ani-jima, Chichi-jima and Haha-jima Islands. Amongst these islands, Chichi-jima and Haha-jima Islands are currently inhabited, whereas Ototo-jima and Ani-jima Islands are uninhabited at present, but were formerly settled by humans. As a result of our surveys, five species of Polydesmida that had not previously been reported from Japan were discovered: *Prosopodesmus jacobsoni* Silvestri, 1910; *P. panporus* Blower & Rundle, 1980 (Haplodesmidae); *Cryptocorypha ornata* (Attems, 1938); *Dyskolonius uniramus* (Attems, 1938) (Pyrgodesmidae); and *Solaenaulus butteli* (Carl, 1922) (Opisotretidae). *P. jacobsoni* was described from Java, Indonesia (Silvestri 1910) and is known to be pantropical through synanthropy (Hoffman 1999). *P. panporus* was described from a hothouse of the Royal Botanic Gardens, Kew, London, UK (Blower and Rundle 1980) and was later recorded from the Cape York Peninsula, Australia, which is considered the likely native range of the species (Mesibov 2012). *C. ornata* was described from Hawaii (Attems 1938b) and is known to be pantropical (Adis et al. 1998). Reexamination of specimens recorded as *Rhipidopeltis sinuata* (currently treated as *Prosopodesmus sinuatus*; see Golovatch et al. (2009b)) from Chichi-jima and Haha-jima Islands by Tanabe (1991) revealed that they belong to this species. *D. uniramus* was described from Vietnam and has not been recorded again since the original description (Attems 1938a). *S. butteli* was described from Sumatra, Indonesia (Carl 1922) and has since been reported from a wide range of localities across Southeast Asia (Golovatch et al. 2010). Takano (1988) recorded *Solaenaulus* sp. with detailed sketches from Chichi-jima Island, which is here regarded as conspecific with this species. We present the morphological basis for the identification of the five species and briefly discuss their distribution, ecology and presumed introductions from other regions.

In addition, a large number of juvenile specimens of *Prosopodesmus* spp. were collected, enabling us to document aspects of their postembryonic development. In this genus, adult males generally have 20 body rings, as observed in *P. jacobsoni*, whereas *P. panporus* is exceptional in having only 19 rings (Blower and Rundle 1980, Mesibov 2012). To investigate the developmental basis of this difference, we compared the formation of the male gonopod primordia during postembryonic development between the two species.

## Materials and methods

**Figure 1.**
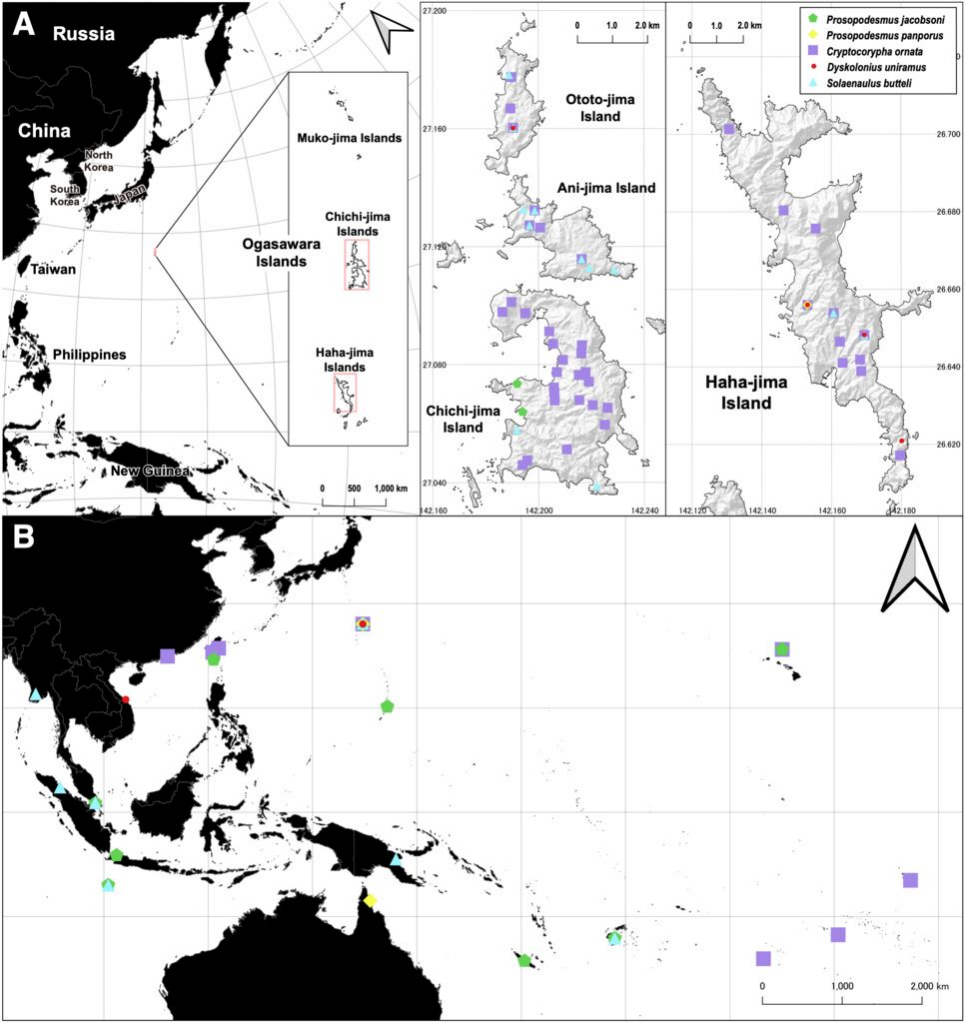
Distribution map of the five newly-recorded millipede species. **A** Map showing the localities of Ototo-jima, Ani-jima, Chichi-jima and Haha-jima Islands in the Ogasawara Islands and the corresponding collection sites of the five species, based on voucher specimens examined in the present study and reexamined materials from Tanabe (1991); **B** Previously known distributions of the five species from East Asia to Oceania, including records from the present study.

Specimens used in this study were collected on Ototo-jima, Ani-jima, Chichi-jima and Haha-jima Islands in the Ogasawara Islands, Japan, from 2023 to 2026 (Fig. 1). Specimens were preserved in 70% or 100% ethanol. Specimens were identified under a stereomicroscope (SZX16; Olympus, Tokyo, Japan). The whole bodies were photographed using a digital camera (EOS 8000D; Canon, Tokyo, Japan), equipped with a macro lens (EF100 mm f/2.8 Macro USM; Canon) and a conversion lens (DCR-250; Raynox, Tokyo, Japan). Photographs were stacked using imaging software (Zerene Stacker; Zerene Systems, Washington, USA). Body length was measured using ImageJ (National Institutes of Health, Bethesda, Maryland, USA), a software programme for measuring segmented line lengths on screen. Distribution maps were generated using Natural Earth (free vector and raster map data) for the broad-scale map and data from the National Land Numerical Information Download Service (Ministry of Land, Infrastructure, Transport and Tourism, Japan) for the Ogasawara Islands. The maps were simplified using Mapshaper (Matthew Bloch, UK) and edited in QGIS Desktop 3.24.1 (QGIS.org).

Fine structures were observed using a scanning electron microscope (JSM-5510LV and JCM-7000; JEOL, Tokyo, Japan). Specimens were dehydrated in 100% ethanol and then air-dried thoroughly at room temperature prior to observation. After examination, the specimens were returned to 70% or 100% ethanol. Some specimens were dehydrated in 100% ethanol, dried using a critical point dryer (Samdri-PVT-3D; Tousimis, Rockville, MD, USA) and sputter-coated (E-1010 Ion Sputter; Hitachi, Tokyo, Japan). These samples were stored, mounted on brass stubs used for SEM observations. Furthermore, observation of the gonopods under transmitted light was performed using a confocal laser scanning microscope (FV3000; Olympus, Tokyo, Japan).

Voucher specimens examined in the present study are deposited in the Kanagawa Prefectural Museum of Natural History (KPM), Japan, under catalogue numbers KPM-NKB 2025–2111. In addition, two specimens previously reported by Tanabe (1991), currently retained in the personal research collection of Tsutomu Tanabe at Kumamoto University, Japan, were reexamined. These specimens remain uncatalogued and are not formally accessioned in an institutional collection. Amongst the materials examined in this study, only representative specimens are detailed in the main text. For a comprehensive list of all examined material, including those not individually listed in the text, please refer to Suppl. material 1.

Specimens from Ototo-jima, Ani-jima and Haha-jima Islands were obtained through the Environment Research and Technology Development Fund (JPMEERF20244RB3) of the Environmental Restoration and Conservation Agency, provided by the Ministry of the Environment, Japan.

## Taxon treatments

### Prosopodesmus jacobsoni

Silvestri, 1910

76037092-133B-5748-9BDC-B3217C124EBA

*Prosopodesmus jacobsoni* Silvestri, 1910 - Silvestri (1910): 362, figs. 6, 7.
*Homodesmus parvus* Chamberlin, 1918 - Chamberlin (1918): 223. Synonymised by Loomis (1950): 166.
*Prosopodesmus jacobsoni hilaris* Brölemann, 1920 - Brölemann (1920): 226, text figs. LXXXI–LXXXII, pl. figs. 134–144. Synonymised by Hoffman (1999): 453.

#### Materials

- **Type status:** Other material. **Occurrence:** catalogNumber: KPM-NKB 2034; occurrenceRemarks: Dissected head, rings 5–7, ring 8, and rings 17–20 of a female adult from ethanol-preserved specimen, mounted on SEM stub.; recordedBy: Yu Hisasue; individualCount: 1; sex: female; lifeStage: adult; associatedOccurrences: derived from catalogNumber: KPM-NKB 2033; occurrenceID: 10F81BAB-2E24-5CE2-A456-6CC48BF416BB; **Taxon:** scientificName: Prosopodesmus jacobsoni Silvestri, 1910; kingdom: Animalia; phylum: Arthropoda; class: Diplopoda; order: Polydesmida; family: Haplodesmidae; genus: Prosopodesmus; specificEpithet: jacobsoni; **Location:** islandGroup: Ogasawara Islands; island: Chichi-jima Island; country: Japan; countryCode: JP; stateProvince: Tokyo; county: Ogasawara; municipality: Futago; verbatimLocality: Futago, Chichi-jima Island, Ogasawara Islands, Tokyo, Japan; decimalLatitude: 27.064038; decimalLongitude: 142.19379; geodeticDatum: WGS84; georeferenceVerificationStatus: verified by collector; **Identification:** identifiedBy: Soma Chiyoda; dateIdentified: 2025–2026; **Event:** samplingProtocol: sifting; eventDate: 2023-12-09; year: 2023; month: 12; day: 9; **Record Level:** institutionCode: KPM; basisOfRecord: PreservedSpecimen
- **Type status:** Other material. **Occurrence:** catalogNumber: KPM-NKB 2035; occurrenceRemarks: Vial contains 3 males (stadium VII), 4 females (stadium VII), 4 males (stadium VI), 6 females (stadium VI), 1 male (stadium V), 1 female (stadium V), and 1 male (stadium IV); body parts of a male juvenile (stadium VII) specimen were dissected and prepared as a separate SEM stub.; recordedBy: Soma Chiyoda; individualCount: 20; sex: mixed sexes; lifeStage: stadia IV–VII juvenile; associatedOccurrences: SEM-derived catalogNumber: KPM-NKB 2036; occurrenceID: 57874F47-0586-5943-B277-014FAFB42554; **Taxon:** scientificName: Prosopodesmus jacobsoni Silvestri, 1910; kingdom: Animalia; phylum: Arthropoda; class: Diplopoda; order: Polydesmida; family: Haplodesmidae; genus: Prosopodesmus; specificEpithet: jacobsoni; **Location:** islandGroup: Ogasawara Islands; island: Chichi-jima Island; country: Japan; countryCode: JP; stateProvince: Tokyo; county: Ogasawara; municipality: Susaki; verbatimLocality: Susaki, Chichi-jima Island, Ogasawara Islands, Tokyo, Japan; decimalLatitude: 27.072318; decimalLongitude: 142.191601; geodeticDatum: WGS84; georeferenceVerificationStatus: verified by collector; **Identification:** identifiedBy: Soma Chiyoda; dateIdentified: 2025–2026; **Event:** eventDate: 2025-02-23; year: 2025; month: 2; day: 23; **Record Level:** institutionCode: KPM; basisOfRecord: PreservedSpecimen
- **Type status:** Other material. **Occurrence:** catalogNumber: KPM-NKB 2039; occurrenceRemarks: Vial contains 1 male (adult), 1 female (adult), 4 males (stadium VII), 1 male (stadium VI), 1 female (stadium VI), 1 male (stadium V), and 1 male (stadium IV).; recordedBy: Yu Hisasue; individualCount: 10; sex: mixed sexes; lifeStage: stadia IV–VII juvenile and adult; occurrenceID: EE7D9729-B541-59AC-A4AB-6F3C58217105; **Taxon:** scientificName: Prosopodesmus jacobsoni Silvestri, 1910; kingdom: Animalia; phylum: Arthropoda; class: Diplopoda; order: Polydesmida; family: Haplodesmidae; genus: Prosopodesmus; specificEpithet: jacobsoni; **Location:** islandGroup: Ogasawara Islands; island: Chichi-jima Island; country: Japan; countryCode: JP; stateProvince: Tokyo; county: Ogasawara; municipality: Susaki; verbatimLocality: Susaki, Chichi-jima Island, Ogasawara Islands, Tokyo, Japan; decimalLatitude: 27.073564; decimalLongitude: 142.191841; geodeticDatum: WGS84; georeferenceVerificationStatus: unverified; georeferenceRemarks: Estimated from locality name; original label lacked GPS coordinates.; **Identification:** identifiedBy: Soma Chiyoda; dateIdentified: 2025–2026; **Event:** samplingProtocol: sifting; eventDate: 2025-02-23; year: 2025; month: 2; day: 23; **Record Level:** institutionCode: KPM; basisOfRecord: PreservedSpecimen
- **Type status:** Other material. **Occurrence:** catalogNumber: KPM-NKB 2040; occurrenceRemarks: Vial contains 2 males (adult), 3 females (adult), and 1 male (stadium VII).; recordedBy: Yu Hisasue; individualCount: 6; sex: mixed sexes; lifeStage: stadium VII juvenile and adult; occurrenceID: 94A7127E-A7F8-595A-A0E7-5CB11C24CF23; **Taxon:** scientificName: Prosopodesmus jacobsoni Silvestri, 1910; kingdom: Animalia; phylum: Arthropoda; class: Diplopoda; order: Polydesmida; family: Haplodesmidae; genus: Prosopodesmus; specificEpithet: jacobsoni; **Location:** islandGroup: Ogasawara Islands; island: Chichi-jima Island; country: Japan; countryCode: JP; stateProvince: Tokyo; county: Ogasawara; municipality: Susaki; verbatimLocality: Susaki, Chichi-jima Island, Ogasawara Islands, Tokyo, Japan; decimalLatitude: 27.0736; decimalLongitude: 142.1919; geodeticDatum: WGS84; georeferenceVerificationStatus: verified by collector; **Identification:** identifiedBy: Soma Chiyoda; dateIdentified: 2025–2026; **Event:** samplingProtocol: sifting; eventDate: 2025-05-10; year: 2025; month: 5; day: 10; **Record Level:** institutionCode: KPM; basisOfRecord: PreservedSpecimen

#### Diagnosis

**Figure 2.**
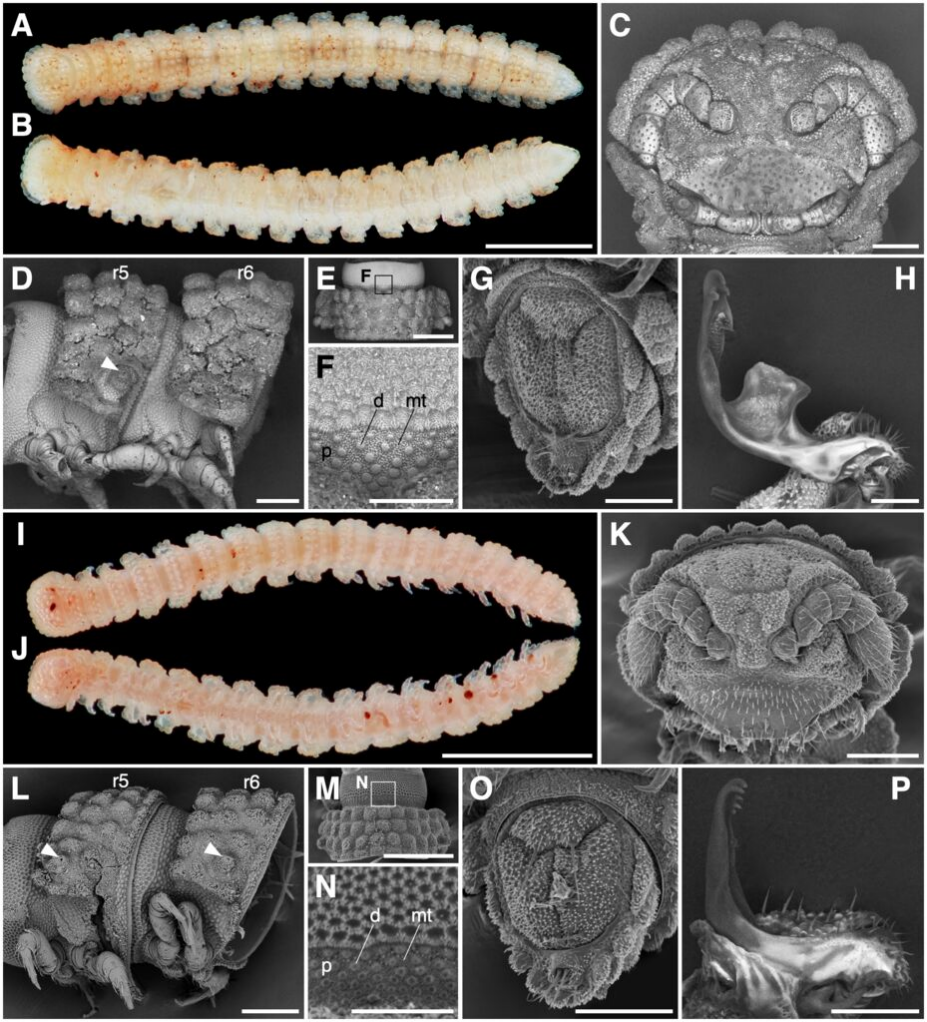
*Prosopodesmus jacobsoni* Silvestri, 1910 from Chichi-jima Island (**A**–**C**: KPM-NKB 2040; **D–F**, **H**: KPM-NKB 2039; **G**: KPM-NKB 2034) and *P. panporus* Blower & Rundle, 1980 from Haha-jima Island (**I**, **J**, **P**: KPM-NKB 2043; **K–O**: KPM-NKB 2044). **A, B** Dorsal view (**A**) and ventral view (**B**) of male adult; **C** Ventral view of head and collum; **D** Lateral view of rings 5, 6; **E** Dorsal view of ring 8; **F** Magnified view of the boxed area in (**E**), showing details of the prozonite surface; **G** Ventral view of telson; **H** Medial view of left gonopod; **I, J** Dorsal view (**I**) and ventral view (**J**) of male adult; **K** Ventral view of head and collum; **L** Lateral view of rings 5, 6; **M** Dorsal view of ring 8; **N** Magnified view of the boxed area in (**M**), showing details of the prozonite surface; **O** Ventral view of telson; **P** Medial view of left gonopod. Arrowheads in (**D, L**) indicate ozopores. Abbreviations: d, disc; mt, microtubercles; p, posterior portion of prozonite; r, ring (e.g. r5 = ring 5). Scale bars: 1 mm (**A, B, I, J**); 200 μm (**E, M**); 100 μm (**C, D, G, K, L, O**); 50 μm (**F, H, N, P**).

**Figure 3.**
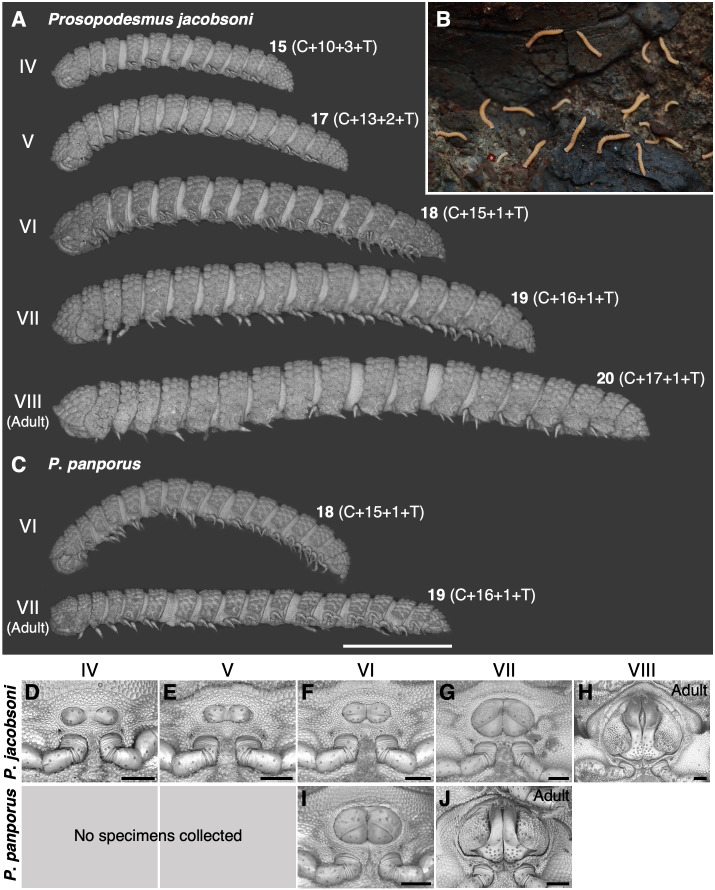
Postembryonic development of *Prosopodesmus* spp. **A** Lateral views of stadium IV–VII juvenile males and an adult male of *P. jacobsoni* (IV–VI: KPM-NKB 2035; VII: KPM-NKB 2039; VIII: KPM-NKB 2040); **B** Various developmental stages of *P. jacobsoni* observed beneath stones, photographed in Susaki, Chichi-jima Island; **C** Lateral views of a stadium VI juvenile male and an adult male of *P. panporus* (KPM-NKB 2043). **A, C** The number of body rings (“C” for collum + podous rings + apodous rings + “T” for telson) is shown to the right of each SEM image; **D–J** Ventral view of ring 7 in males, showing gonopod primordia or fully formed gonopods: **D–H**, *P. jacobsoni* (**D**–**F**: KPM-NKB 2035; **G**: KPM-NKB 2039; **H**: KPM-NKB 2040); **I, J**, *P. panporus* (KPM-NKB 2043). Scale bars: 1 mm (**A, C**); 50 μm (**D–J**).

Based on the descriptions by Silvestri (1910) and Blower and Rundle (1980), the specimens were identified as *P. jacobsoni* by the following characters: males with head + 20 rings, 5.3–5.7 mm long (Fig. 2A, B and Fig. 3A); female with head + 20 rings, 5.4–5.8 mm long; colour yellowish-brown (Fig. 2A, B and Fig. 3B); anterior edge of collum with 12 lobes (Fig. 2C); ozopores on rings 5, 7–19 (Fig. 2D); posterior portion of prozonite with small discs and microtubercles (Fig. 2E and F); posterior edge of telson with 10 lobes (Fig. 2G); gonopod telopodite bent posteriorly at mid-length, with large lateral lamella on proximal half of telopodite, two subapical teeth, a small projection bearing fine setae slightly proximal to the teeth (Fig. 2H and Fig. 3H).

#### Distribution

The mainland United States (Florida, Louisiana), Jamaica, Haiti, Puerto Rico, Virgin Islands, St. Eustatius, Panama, Brazil, Zanzibar, India, Singapore, Indonesia (Java; type locality: “Batavia (Giava)”), Christmas Island (Indian Ocean), New Caledonia, Fiji, Galapagos Islands, Hawaiian Islands, Saipan, Taiwan, Japan (Chichi-jima Island) (Silvestri 1910, Chamberlin 1945, Hoffman 1999, Shelley and Golovatch 2000, Jeekel 2006, Akkari and Enghoff 2011, Golovatch et al. 2011, Decker 2013, present study) (Fig. 1).

#### Ecology

This species was collected from under fallen logs and stones in forests, as well as from leaf litter accumulated amongst rocks near the coast (Fig. 3B). Silvestri (1910) collected this species from an ant nest, but in the present study, it was not found in association with ants. Susaki on Chichi-jima Island, where numerous specimens of this species were collected, was reclaimed as a military site in the 1930s (Kamijo 2010) and the area is still considered to be a frequently disturbed environment.

#### Notes

##### Japanese name

We herein propose the new Japanese name “Yakobuson-kamayasude” (Japanese: ヤコブソンカマヤスデ) for this species. This Japanese name is derived from the specific epithet of this species.

#### Postembryonic development

Individuals representing each developmental stage from stadium IV juvenile to the adult were collected (Fig. 3A and B). Developmental stadia were determined, based on the number of body rings, following the postembryonic developmental pattern of *P. panporus* described by Blower and Rundle (1980). The number of body rings, leg pairs and body length at each stadium are summarised in Table 2.

In all five stadia examined, from stadium IV juveniles to adults, a pair of gonopod primordia or fully developed gonopods was present on the ventral side of ring 7 in males (Fig. 3D–H). In stadium IV juveniles, the gonopod primordia were papillary, each slightly smaller in diameter than the coxa of the walking leg on ring 7. The left and right primordia showed no medial contact (Fig. 3D). In stadium V juveniles, the gonopod primordia remained papillary and were still slightly smaller than the coxa of the leg on ring 7. The left and right primordia were in slight medial contact (Fig. 3E). In stadium VI juveniles, the gonopod primordia became irregularly circular, each approximately equal in diameter to the coxa of the leg on ring 7. A weak transverse groove was present laterally and the left and right primordia were in medial contact (Fig. 3F). In stadium VII juveniles, the gonopod primordia were semicircular, each approximately 1.5 times wider than the diameter of the coxa of the leg on ring 7. Each primordium was divided into anterior and posterior regions by an oblique groove; the anterior region with a rough surface, whereas the posterior region was smooth. The left and right primordia were in medial contact and together formed an elliptical structure (Fig. 3G).

In adults, the gonopods were semicircular in ventral view, each slightly exceeding twice the diameter of the coxa of the leg on ring 7 in width. The coxa exhibited a coarse, reticulate surface texture and bore a well-developed telopodite on the medial side. The telopodite had a smooth surface with dense setae at its base. The left and right gonopods were in medial contact, together forming an almost circular structure (Fig. 2H and Fig. 3H).

### Prosopodesmus panporus

Blower & Rundle, 1980

176149DA-D2B8-5A42-9791-17B0D7B3B702

*Prosopodesmus panporus* Blower & Rundle, 1980 - Blower and Rundle (1980): 27, figs. 1–3, 6–8, table 1.

#### Materials

- **Type status:** Other material. **Occurrence:** catalogNumber: KPM-NKB 2043; occurrenceRemarks: Vial contains 2 males (adult), 1 female (adult), and 1 male (stadium VI); body parts of a male adult specimen were dissected and prepared as a separate SEM stub.; recordedBy: Soma Chiyoda; individualCount: 4; sex: mixed sexes; lifeStage: stadium VI juvenile and adult; associatedOccurrences: SEM-derived catalogNumber: KPM-NKB 2044; occurrenceID: 451D9C6E-2F07-5BA0-979B-11D27EA27EF5; **Taxon:** scientificName: Prosopodesmus panporus Blower & Rundle, 1980; kingdom: Animalia; phylum: Arthropoda; class: Diplopoda; order: Polydesmida; family: Haplodesmidae; genus: Prosopodesmus; specificEpithet: panporus; **Location:** islandGroup: Ogasawara Islands; island: Haha-jima Island; country: Japan; countryCode: JP; stateProvince: Tokyo; county: Ogasawara; municipality: Nishiura; verbatimLocality: Nishiura, Haha-jima Island, Ogasawara Islands, Tokyo, Japan; decimalLatitude: 26.656034; decimalLongitude: 142.153019; geodeticDatum: WGS84; georeferenceVerificationStatus: unverified; georeferenceRemarks: Estimated from locality name; original label lacked GPS coordinates.; **Identification:** identifiedBy: Soma Chiyoda; dateIdentified: 2025–2026; **Event:** eventDate: 2025-02-19; year: 2025; month: 2; day: 19; **Record Level:** institutionCode: KPM; basisOfRecord: PreservedSpecimen
- **Type status:** Other material. **Occurrence:** catalogNumber: KPM-NKB 2044; occurrenceRemarks: Dissected head, rings 5–6, ring 8, rings 16–19, and right gonopod of a male adult from ethanol-preserved specimen, mounted on SEM stub.; recordedBy: Soma Chiyoda; individualCount: 1; sex: male; lifeStage: adult; associatedOccurrences: derived from catalogNumber: KPM-NKB 2043; occurrenceID: 80AF6461-E76C-5396-84DB-393CAEF56BB2; **Taxon:** scientificName: Prosopodesmus panporus Blower & Rundle, 1980; kingdom: Animalia; phylum: Arthropoda; class: Diplopoda; order: Polydesmida; family: Haplodesmidae; genus: Prosopodesmus; specificEpithet: panporus; **Location:** islandGroup: Ogasawara Islands; island: Haha-jima Island; country: Japan; countryCode: JP; stateProvince: Tokyo; county: Ogasawara; municipality: Nishiura; verbatimLocality: Nishiura, Haha-jima Island, Ogasawara Islands, Tokyo, Japan; decimalLatitude: 26.656034; decimalLongitude: 142.153019; geodeticDatum: WGS84; georeferenceVerificationStatus: unverified; georeferenceRemarks: Estimated from locality name; original label lacked GPS coordinates.; **Identification:** identifiedBy: Soma Chiyoda; dateIdentified: 2025–2026; **Event:** eventDate: 2025-02-19; year: 2025; month: 2; day: 19; **Record Level:** institutionCode: KPM; basisOfRecord: PreservedSpecimen

#### Diagnosis

Based on the descriptions by Blower and Rundle (1980) and Mesibov (2012), the specimens were identified as *P. panporus* by the following characters: males with head + 19 rings, 3.5–3.7 mm long (Fig. 2I, J and Fig. 3C); female with head + 20 rings, 3.5 mm long; colour pale pinkish-beige (Fig. 2I and J); anterior edge of collum with 12 lobes (Fig. 2K); ozopores on all podous rings, rings 5–17 in the male and rings 5–18 in the female (Fig. 2L); posterior portion of prozonite with small discs and microtubercles (Fig. 2M and N); posterior edge of telson with 12 lobes, although it should be noted that some of them are small and not clearly distinguishable (Fig. 2O); gonopod telopodite bent posteriorly at mid-length, lateral edge near tip with several rounded teeth (Fig. 2P and Fig. 3J).

#### Distribution

United Kingdom (Royal Botanic Gardens, Kew), Australia (Queensland), Japan (Haha-jima Island) (Blower and Rundle 1980, Mesibov 2012, present study) (Fig. 1).

#### Ecology

This species was collected from beneath fallen logs in a forest. Although the collection site was located near a paved road, it was within a forested area far from settlements and the port.

#### Notes

##### Japanese name

We herein propose the new Japanese name “Fushikake-kamayasude” (Japanese: フシカケカマヤスデ) for this species. This Japanese name refers to the fact that adult males of this species have 19 rings, one fewer than in other congeners.

#### Postembryonic development

A male stadium VI juvenile, an adult male and an adult female were collected (Fig. 3C). Developmental stadia were determined, based on the number of body rings, following the postembryonic developmental pattern of this species described by Blower and Rundle (1980). The numbers of body rings, leg pairs and body length at each stadium are summarised in Table 2. On the ventral side of ring 7, a pair of gonopod primordia and fully developed gonopods were observed in the male stadium VI juvenile and adult, respectively (Fig. 3I and J).

In the stadium VI juvenile, the gonopod primordia were semicircular, each slightly wider than the diameter of the coxa of the leg on ring 7. Each primordium was divided into anterior and posterior regions by an oblique groove; the anterior region had a weakly wrinkled surface, whereas the posterior region was smooth. A very short projection was present on the medial side of the anterior region, with the left and right projections slightly overlapping. The left and right primordia were in medial contact and together formed an elliptical structure (Fig. 3I).

In the adult, the gonopods were semicircular in ventral view, each approximately twice as wide as the diameter of the coxa of the leg on ring 7. The coxa exhibited a coarse, reticulate surface texture and bore a well-developed telopodite on its medial side. The telopodite had a smooth surface with dense setae at its base. The left and right gonopods were in medial contact, together forming an elliptical structure (Fig. 2P and Fig. 3J).

### Cryptocorypha ornata

(Attems, 1938)

0338F64C-5F59-550B-A329-8B34C0CF03CD

*Platykapelus ornatus* Attems, 1938 - Attems (1938b): 379–382, figs. 14–20.
*Cryptocorypha ornata* (Attems, 1938) - Hoffman (1977): 374–376, fig. 144. Combination proposed by Hoffman (1977).

#### Materials

- **Type status:** Other material. **Occurrence:** catalogNumber: KPM-NKB 2053; recordedBy: Yu Hisasue; individualCount: 2; sex: female; lifeStage: adult; occurrenceID: D4792B29-9168-5D62-805B-D6BD9430209D; **Taxon:** scientificName: Cryptocorypha ornata (Attems, 1938); kingdom: Animalia; phylum: Arthropoda; class: Diplopoda; order: Polydesmida; family: Pyrgodesmidae; genus: Cryptocorypha; specificEpithet: ornata; **Location:** islandGroup: Ogasawara Islands; island: Chichi-jima Island; country: Japan; countryCode: JP; stateProvince: Tokyo; county: Ogasawara; locality: Mt. Chuo-san; verbatimLocality: Mt. Chuo-san, Chichi-jima Island, Ogasawara Islands, Tokyo, Japan; decimalLatitude: 27.074258; decimalLongitude: 142.218896; geodeticDatum: WGS84; georeferenceVerificationStatus: verified by collector; **Identification:** identifiedBy: Soma Chiyoda; dateIdentified: 2025–2026; **Event:** samplingProtocol: sifting; eventDate: 2024-09-22; year: 2024; month: 9; day: 22; **Record Level:** institutionCode: KPM; basisOfRecord: PreservedSpecimen
- **Type status:** Other material. **Occurrence:** catalogNumber: KPM-NKB 2056; recordedBy: Soma Chiyoda; individualCount: 2; sex: male; lifeStage: adult; occurrenceID: 1D95AD2A-7A71-5B9A-A631-CEBB900C6F7A; **Taxon:** scientificName: Cryptocorypha ornata (Attems, 1938); kingdom: Animalia; phylum: Arthropoda; class: Diplopoda; order: Polydesmida; family: Pyrgodesmidae; genus: Cryptocorypha; specificEpithet: ornata; **Location:** islandGroup: Ogasawara Islands; island: Haha-jima Island; country: Japan; countryCode: JP; stateProvince: Tokyo; county: Ogasawara; municipality: Nishiura; verbatimLocality: Nishiura, Haha-jima Island, Ogasawara Islands, Tokyo, Japan; decimalLatitude: 26.656034; decimalLongitude: 142.153019; geodeticDatum: WGS84; georeferenceVerificationStatus: unverified; georeferenceRemarks: Estimated from locality name; original label lacked GPS coordinates.; **Identification:** identifiedBy: Soma Chiyoda; dateIdentified: 2025–2026; **Event:** eventDate: 2025-02-19; year: 2025; month: 2; day: 19; **Record Level:** institutionCode: KPM; basisOfRecord: PreservedSpecimen
- **Type status:** Other material. **Occurrence:** catalogNumber: KPM-NKB 2058; occurrenceRemarks: Vial contains 2 males (adult) and 1 female (adult).; recordedBy: Soma Chiyoda; individualCount: 3; sex: mixed sexes; lifeStage: adult; occurrenceID: 017B68DB-746C-5894-8821-A635C808BC09; **Taxon:** scientificName: Cryptocorypha ornata (Attems, 1938); kingdom: Animalia; phylum: Arthropoda; class: Diplopoda; order: Polydesmida; family: Pyrgodesmidae; genus: Cryptocorypha; specificEpithet: ornata; **Location:** islandGroup: Ogasawara Islands; island: Haha-jima Island; country: Japan; countryCode: JP; stateProvince: Tokyo; county: Ogasawara; municipality: Funakiyama; verbatimLocality: Funakiyama, Haha-jima Island, Ogasawara Islands, Tokyo, Japan; decimalLatitude: 26.641897; decimalLongitude: 142.168099; geodeticDatum: WGS84; georeferenceVerificationStatus: verified by collector; **Identification:** identifiedBy: Soma Chiyoda; dateIdentified: 2025–2026; **Event:** eventDate: 2025-02-21; year: 2025; month: 2; day: 21; **Record Level:** institutionCode: KPM; basisOfRecord: PreservedSpecimen
- **Type status:** Other material. **Occurrence:** catalogNumber: KPM-NKB 2064; occurrenceRemarks: Vial contains 7 males (adult), 6 females (adult), and 1 female (stadium VII).; recordedBy: Yu Hisasue; individualCount: 14; sex: mixed sexes; lifeStage: stadium VII juvenile and adult; occurrenceID: FE490685-BF7F-599F-ABD3-837DDD2C86E6; **Taxon:** scientificName: Cryptocorypha ornata (Attems, 1938); kingdom: Animalia; phylum: Arthropoda; class: Diplopoda; order: Polydesmida; family: Pyrgodesmidae; genus: Cryptocorypha; specificEpithet: ornata; **Location:** islandGroup: Ogasawara Islands; island: Chichi-jima Island; country: Japan; countryCode: JP; stateProvince: Tokyo; county: Ogasawara; municipality: Otaki; verbatimLocality: Otaki, Chichi-jima Island, Ogasawara Islands, Tokyo, Japan; decimalLatitude: 27.0663; decimalLongitude: 142.2204; geodeticDatum: WGS84; georeferenceVerificationStatus: verified by collector; **Identification:** identifiedBy: Soma Chiyoda; dateIdentified: 2025–2026; **Event:** samplingProtocol: sifting; eventDate: 2025-04-06; year: 2025; month: 4; day: 6; **Record Level:** institutionCode: KPM; basisOfRecord: PreservedSpecimen
- **Type status:** Other material. **Occurrence:** catalogNumber: KPM-NKB 2093; occurrenceRemarks: Vial contains 3 females (adult), 1 female (stadium VII), and 1 female (stadium VI).; recordedBy: Yu Hisasue; individualCount: 5; sex: female; lifeStage: stadia VI–VII juvenile and adult; occurrenceID: 572D7C82-0293-5268-AED6-D1D28808038E; **Taxon:** scientificName: Cryptocorypha ornata (Attems, 1938); kingdom: Animalia; phylum: Arthropoda; class: Diplopoda; order: Polydesmida; family: Pyrgodesmidae; genus: Cryptocorypha; specificEpithet: ornata; **Location:** islandGroup: Ogasawara Islands; island: Chichi-jima Island; country: Japan; countryCode: JP; stateProvince: Tokyo; county: Ogasawara; locality: Mt. Ogami-yama; verbatimLocality: Mt. Ogami-yama, Chichi-jima Island, Ogasawara Islands, Tokyo, Japan; decimalLatitude: 27.0975; decimalLongitude: 142.1949; geodeticDatum: WGS84; georeferenceVerificationStatus: verified by collector; **Identification:** identifiedBy: Soma Chiyoda; dateIdentified: 2025–2026; **Event:** samplingProtocol: sifting; eventDate: 2025-11-23; year: 2025; month: 11; day: 23; **Record Level:** institutionCode: KPM; basisOfRecord: PreservedSpecimen
- **Type status:** Other material. **Occurrence:** catalogNumber: TT-Oga-001; recordedBy: Kiyonori Tomiyama; individualCount: 2; sex: male; lifeStage: adult; previousIdentifications: Rhipidopeltis sinuata Miyosi (Tanabe 1991); occurrenceID: ED3A1CC6-5709-59C2-B2CE-6E9708788AC7; **Taxon:** scientificName: Cryptocorypha ornata (Attems, 1938); kingdom: Animalia; phylum: Arthropoda; class: Diplopoda; order: Polydesmida; family: Pyrgodesmidae; genus: Cryptocorypha; specificEpithet: ornata; **Location:** islandGroup: Ogasawara Islands; island: Chichi-jima Island; country: Japan; countryCode: JP; stateProvince: Tokyo; county: Ogasawara; municipality: Higashikaigan; verbatimLocality: Higashikaigan, Chichi-jima Island, Ogasawara Islands, Tokyo, Japan; decimalLatitude: 27.0654; decimalLongitude: 142.226; geodeticDatum: WGS84; georeferenceVerificationStatus: unverified; georeferenceRemarks: Estimated from locality name; original label lacked GPS coordinates.; **Identification:** identifiedBy: Soma Chiyoda; dateIdentified: 2025–2026; **Event:** eventDate: 1990-03-24; year: 1990; month: 3; day: 24; **Record Level:** institutionCode: Personal collection; collectionCode: Tsutomu Tanabe; ownerInstitutionCode: Tsutomu Tanabe; basisOfRecord: PreservedSpecimen
- **Type status:** Other material. **Occurrence:** catalogNumber: TT-Oga-002; recordedBy: Hasegawa; individualCount: 1; sex: male; lifeStage: adult; previousIdentifications: Rhipidopeltis sinuata Miyosi (Tanabe 1991); occurrenceID: 43A8052B-A84D-5D4D-8B9F-3D39D8C06681; **Taxon:** scientificName: Cryptocorypha ornata (Attems, 1938); kingdom: Animalia; phylum: Arthropoda; class: Diplopoda; order: Polydesmida; family: Pyrgodesmidae; genus: Cryptocorypha; specificEpithet: ornata; **Location:** islandGroup: Ogasawara Islands; island: Haha-jima Island; country: Japan; countryCode: JP; stateProvince: Tokyo; county: Ogasawara; locality: Front of Chibusayama Dam; verbatimLocality: Front of Chibusayama Dam, Haha-jima Island, Ogasawara Islands, Tokyo, Japan; decimalLatitude: 26.646539; decimalLongitude: 142.162297; geodeticDatum: WGS84; georeferenceVerificationStatus: unverified; georeferenceRemarks: Estimated from locality name; original label lacked GPS coordinates.; **Identification:** identifiedBy: Soma Chiyoda; dateIdentified: 2025–2026; **Event:** eventDate: 1990-10-12; year: 1990; month: 10; day: 12; **Record Level:** institutionCode: Personal collection; collectionCode: Tsutomu Tanabe; ownerInstitutionCode: Tsutomu Tanabe; basisOfRecord: PreservedSpecimen

#### Diagnosis

**Figure 4.**
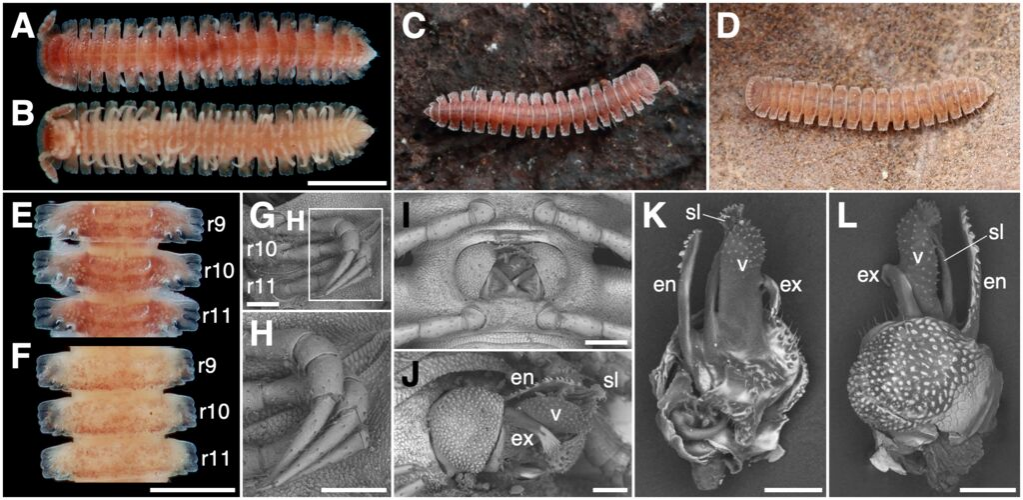
*Cryptocorypha ornata* (Attems, 1938) from Chichi-jima and Haha-jima Islands. **A, B** Dorsal view (**A**) and ventral view (**B**) of male adult (KPM-NKB 2056) **C** Male adult in life, photographed in Funamidai, Haha-jima Island; **D** Female adult in life, photographed in Mt. Ogami-yama, Chichi-jima Island (KPM-NKB 2093); **E, F** Dorsal view of rings 9–11 of male adult (**E**; KPM-NKB 2058) and female adult (**F**; KPM-NKB 2053); **G** Left walking legs of ring 10 (Tanabe collection, re-examined material, Higashikaigan, Chichi-jima Island); **H** Magnified view of the boxed area in (**G**), showing details of last tibiae; **I** Ventral view of intact gonopods (KPM-NKB 2056); **J** Ventrolateral view of intact right gonopod (Tanabe collection, reexamined material, Higashikaigan, Chichi-jima Island); **K, L** Medial (**K**) and lateral (**L**) view of left gonopod (KPM-NKB 2064). Abbreviations: en, endomere; ex, exomere; r, ring (e.g. r9 = ring 9); sl, solenomere; v, velum. Scale bars: 1 mm (**A, B**); 500 μm (**E, F**); 100 μm (**G–I**); 50 μm (**J–L**).

Based on the provisional key to the genus provided by Golovatch (2019), the specimens were identified as *C. ornata* by the following characters: males with head + 20 rings, 4.2–5.0 mm long (Fig. 4A and B); female with head + 20 rings, 4.7–6.1 mm long; body considerably slender (Fig. 4A–D); male colour dull pink, female colour slightly paler (Fig. 4A–F); the last tibiae bear apicodorsal setae, but their bases are not columnar and thus cannot be regarded as trichosteles (Fig. 4G and H); gonopodal telopodite quadripartite: an apically plumose and slender solenomere only very slightly higher than a very prominent and densely spiculate velum and a distomesally slightly spinulate and slender endomere, while exomere a curved, thick, distally denticulate, lateral process (Fig. 4I–L).

#### Distribution

The mainland United States (Georgia), Saint Helena, Marquesas Islands, Tahiti, Cook Islands, Hawaiian Islands (type locality: “Nanhi Goulch, Mauna Kea, Hawai”), Hong Kong, Taiwan, Japan (Ototo-jima Island, Ani-jima Island, Chichi-jima Island, Haha-jima Island) (Attems 1938b, Tanabe 1991, Adis et al. 1998, Golovatch et al. 2011, Andrew et al. 2024, present study) (Fig. 1).

#### Ecology

This species was collected from beneath fallen logs and within leaf litter in both forest interiors and edges. It was found across a wide range of habitats, from frequently disturbed, dry environments near agricultural fields and coastal areas to relatively undisturbed, well-preserved forests.

#### Notes

##### Japanese name

We herein propose the new Japanese name “Hoso-ôgiyasude” (Japanese: ホソオウギヤスデ) for this species. This Japanese name is derived from the relatively slender body of this species compared with congeners already known from Japan.

##### Reexamination of specimens reported by Tanabe (1991)

Reexamination of specimens reported as *Prosopodesmus sinuatus* by Tanabe (1991) from Chichi-jima and Haha-jima Islands revealed that they belong to *Cryptocorypha ornata* (Fig. 4G, H and J). The specimens, which are preserved in the personal research collection of Tsutomu Tanabe, were directly examined in the present study.

*C. ornata* resembles *P. sinuatus* in having the head largely concealed by the collum, well-developed paranota and distinct basal notches on the posterior margins of the paranota (Fig. 4A–F; Miyosi 1958). However, the gonopods differ markedly between the two species and provide decisive diagnostic characters. In the examined specimens, the gonopod morphology fully agrees with that described for *C. ornata* and clearly differs from that of *P. sinuatus*. Additional characters further support this identification. In *C. ornata*, the collum is divided into 12 lobes, whereas *P. sinuatus* possesses 14 lobes (Miyosi 1958). Furthermore, the dorsal profile in *C. ornata* is smoothly continuous, whereas *P. sinuatus* exhibits a strongly arched trunk distinctly demarcated from the paranota (Miyosi 1958). These consistent differences confirm that the specimens previously identified as *P. sinuatus* by Tanabe (1991) were misidentified and should be referred to *C. ornata*.

Accordingly, *P. sinuatus* is excluded from the confirmed millipede fauna of the Ogasawara Islands and *C. ornata* is established as the species represented by the material reported in Tanabe (1991).

### Dyskolonius uniramus

(Attems, 1938)

E86E278E-BB0E-5C19-9517-AF0E90607C89

Steganostigmus (Dyskolonius) uniramus Attems, 1938 - Attems (1938a): 243–244, figs. 80–86.

#### Materials

- **Type status:** Other material. **Occurrence:** catalogNumber: KPM-NKB 2025; recordedBy: Soma Chiyoda; individualCount: 1; sex: female; lifeStage: stadium VII juvenile; occurrenceID: BFE454F0-E72E-57D9-878F-C26E835CB357; **Taxon:** scientificName: Dyskolonius uniramus (Attems, 1938); kingdom: Animalia; phylum: Arthropoda; class: Diplopoda; order: Polydesmida; family: Pyrgodesmidae; genus: Dyskolonius; specificEpithet: uniramus; **Location:** islandGroup: Ogasawara Islands; island: Haha-jima Island; country: Japan; countryCode: JP; stateProvince: Tokyo; county: Ogasawara; municipality: Nishiura; verbatimLocality: Nishiura, Haha-jima Island, Ogasawara Islands, Tokyo, Japan; decimalLatitude: 26.656034; decimalLongitude: 142.153019; geodeticDatum: WGS84; georeferenceVerificationStatus: unverified; georeferenceRemarks: Estimated from locality name; original label lacked GPS coordinates.; **Identification:** identifiedBy: Soma Chiyoda; dateIdentified: 2025–2026; **Event:** eventDate: 2025-02-19; year: 2025; month: 2; day: 19; **Record Level:** institutionCode: KPM; basisOfRecord: PreservedSpecimen
- **Type status:** Other material. **Occurrence:** catalogNumber: KPM-NKB 2027; occurrenceRemarks: Body parts of the specimen were dissected and prepared as a separate SEM stub.; recordedBy: Soma Chiyoda; individualCount: 1; sex: male; lifeStage: adult; associatedOccurrences: SEM-derived catalogNumbers: KPM-NKB 2028, KPM-NKB 2029; occurrenceID: 28B8D8CF-59F9-5975-881D-60D0E376DCCD; **Taxon:** scientificName: Dyskolonius uniramus (Attems, 1938); kingdom: Animalia; phylum: Arthropoda; class: Diplopoda; order: Polydesmida; family: Pyrgodesmidae; genus: Dyskolonius; specificEpithet: uniramus; **Location:** islandGroup: Ogasawara Islands; island: Haha-jima Island; country: Japan; countryCode: JP; stateProvince: Tokyo; county: Ogasawara; municipality: Funakiyama; verbatimLocality: Funakiyama, Haha-jima Island, Ogasawara Islands, Tokyo, Japan; decimalLatitude: 26.648271; decimalLongitude: 142.169293; geodeticDatum: WGS84; georeferenceVerificationStatus: unverified; georeferenceRemarks: Estimated from locality name; original label lacked GPS coordinates.; **Identification:** identifiedBy: Soma Chiyoda; dateIdentified: 2025–2026; **Event:** eventDate: 2025-02-21; year: 2025; month: 2; day: 21; **Record Level:** institutionCode: KPM; basisOfRecord: PreservedSpecimen
- **Type status:** Other material. **Occurrence:** catalogNumber: KPM-NKB 2029; occurrenceRemarks: Dissected head, rings 5–6, ring 8, and rings 17–20 from ethanol-preserved specimen, mounted on SEM stub.; recordedBy: Soma Chiyoda; individualCount: 1; sex: male; lifeStage: adult; associatedOccurrences: derived from catalogNumber: KPM-NKB 2027; occurrenceID: ADA3EEF1-0D17-5E15-BDBF-8D8407831221; **Taxon:** scientificName: Dyskolonius uniramus (Attems, 1938); kingdom: Animalia; phylum: Arthropoda; class: Diplopoda; order: Polydesmida; family: Pyrgodesmidae; genus: Dyskolonius; specificEpithet: uniramus; **Location:** islandGroup: Ogasawara Islands; island: Haha-jima Island; country: Japan; countryCode: JP; stateProvince: Tokyo; county: Ogasawara; municipality: Funakiyama; verbatimLocality: Funakiyama, Haha-jima Island, Ogasawara Islands, Tokyo, Japan; decimalLatitude: 26.648271; decimalLongitude: 142.169293; geodeticDatum: WGS84; georeferenceVerificationStatus: unverified; georeferenceRemarks: Estimated from locality name; original label lacked GPS coordinates.; **Identification:** identifiedBy: Soma Chiyoda; dateIdentified: 2025–2026; **Event:** eventDate: 2025-02-21; year: 2025; month: 2; day: 21; **Record Level:** institutionCode: KPM; basisOfRecord: PreservedSpecimen
- **Type status:** Other material. **Occurrence:** catalogNumber: KPM-NKB 2031; recordedBy: Yu Hisasue; individualCount: 1; sex: male; lifeStage: adult; occurrenceID: B50672AD-847F-57B9-87A9-E6D0FA882D8C; **Taxon:** scientificName: Dyskolonius uniramus (Attems, 1938); kingdom: Animalia; phylum: Arthropoda; class: Diplopoda; order: Polydesmida; family: Pyrgodesmidae; genus: Dyskolonius; specificEpithet: uniramus; **Location:** islandGroup: Ogasawara Islands; island: Ototo-jima Island; country: Japan; countryCode: JP; stateProvince: Tokyo; county: Ogasawara; locality: Ai-no-sawa; verbatimLocality: Ai-no-sawa, Ototo-jima Island, Ogasawara Islands, Tokyo, Japan; decimalLatitude: 27.1603; decimalLongitude: 142.1904; geodeticDatum: WGS84; georeferenceVerificationStatus: verified by collector; **Identification:** identifiedBy: Soma Chiyoda; dateIdentified: 2025–2026; **Event:** samplingProtocol: sifting; eventDate: 2025-08-23; year: 2025; month: 8; day: 23; **Record Level:** institutionCode: KPM; basisOfRecord: PreservedSpecimen

#### Diagnosis

**Figure 5.**
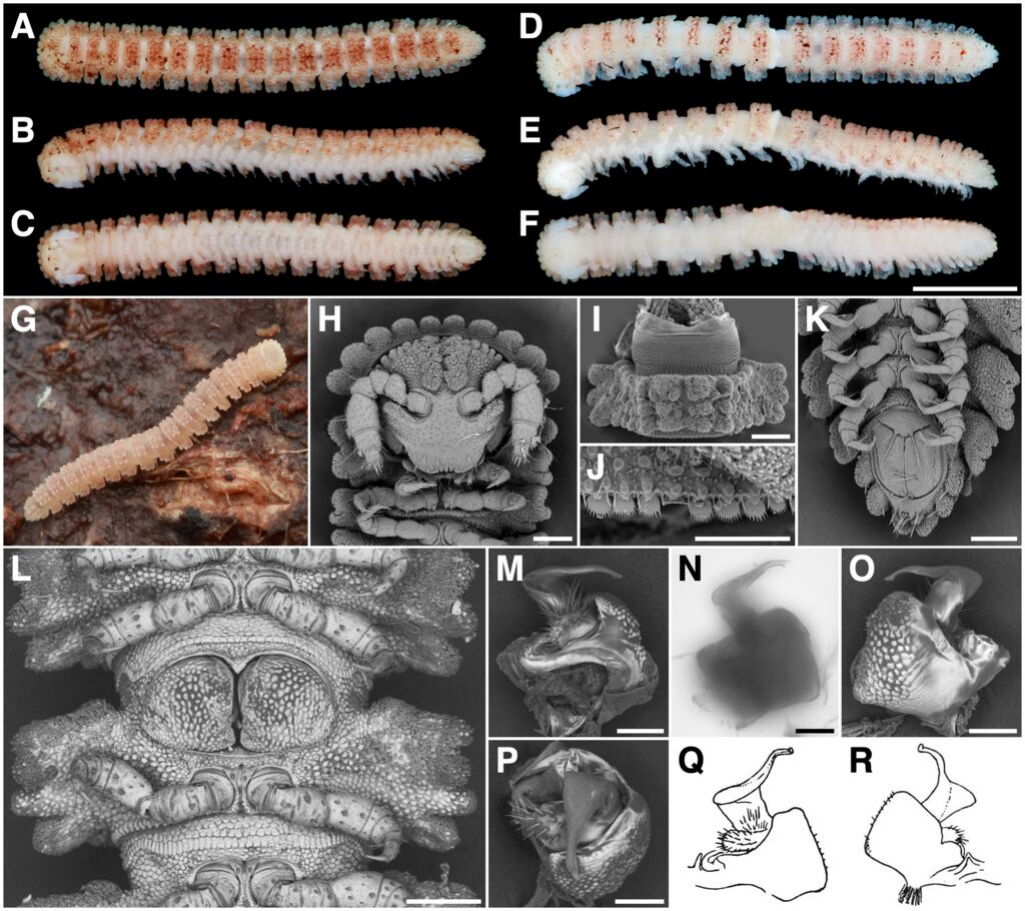
*Dyskolonius uniramus* (Attems, 1938) from Ototo-jima (**A–C, L**: KPM-NKB 2031) and Haha-jima Islands (**D–F, M**–**P**: KPM-NKB 2027; **G**: KPM-NKB 2025; **H**–**K**: KPM-NKB 2029). **A–F** Dorsal view (**A, D**), lateral view (**B, E**) and ventral view (**C, F**) of male adult; **G** Female, stadium VII juvenile observed beneath a fallen log, in life, photographed in Nishiura, Haha-jima Island (KPM-NKB 2025); **H** Ventral view of head and collum; **I** Dorsal view of ring 8; **J** Posterior margin of ring 6; **K** Ventral view of telson; **L** Ventral view of intact gonopods; **M–P** Medial (**M, N**), lateral (**O**) and ventral (**P**) view of left gonopod. SEM images (**M, O, P**) and transmitted light image (**N**); **Q, R** Medial (**Q**) and lateral view (**R**) of left gonopod, reproduced from Attems (1938a), p.244. Scale bars: 1 mm (**A–F**); 100 μm (**H, I, K, L**); 50 μm (**J, M–P**).

Based on the original description by Attems (1938a), the specimens were identified as *D. uniramus* by the following characters: male with head + 20 rings, 4.0–4.4 mm long, slightly smaller than described in the original description (Fig. 5A–F); colour pale pinkish-beige to brown (Fig. 5A, B, D, E and G); anterior edge of collum with 10 lobes (Fig. 5H); paranota situated somewhat low, with the anterior margin projecting shoulder-like and the lateral margin divided into three or four rounded incisions (Fig. 5A, D, G and I); a row of small, round plates with finely fringed margins and two setae at the base along the posterior margin of each metazonite (Fig. 5J); posterior edge of telson with six large tubercles arranged in a semicircle, with the terminal setae displaced ventrally and completely concealed by these six tubercles (Fig. 5K); gonopod coxa large, outer surface partly verrucose, with short setae sparsely distributed (Fig. 5L–P); basal part of gonopod telopodite densely setose, continuing into a broad lamellar portion strongly curved posteriorly; the distal part of the telopodite tapering gradually, slightly curved and bearing the seminal groove (Fig. 5M–P). The gonopod telopodite appears more strongly bent in the SEM images than in the original description, but this is likely an artefact caused by drying of the sample. As confirmed from transmitted-light observations, the actual morphology of the present material agrees well with the figures in the original description (Fig. 5M–R).

#### Distribution

Vietnam, Japan (Ototo-jima Island, Haha-jima Island) (Attems 1938a, present study) (Fig. 1).

#### Ecology

This species was collected from beneath fallen logs in relatively undisturbed, well-preserved forests (Fig. 5G).

#### Taxon discussion

The species was originally described as Steganostigmus (Dyskolonius) uniramus by Attems (1938a). Later, *Dyskolonius* was elevated from subgenus to genus rank by Hoffman (1980), which resulted in the automatic new combination *Dyskolonius uniramus*, even though Hoffman did not explicitly state the recombination (Enghoff et al. 2004).

#### Notes

##### Japanese name

We herein propose the new Japanese name “Chôemon-yasude” (Japanese: チョウエモンヤスデ) for this species and “Chôemon-yasude-zoku” (Japanese: チョウエモンヤスデ属) for the genus *Dyskolonius*. This Japanese name is derived from Chôemon, one of the first Japanese people to land on Haha-jima Island in the Ogasawara Islands in 1670.

### Solaenaulus butteli

(Carl, 1922)

D84166EB-A4A2-57C5-9C91-95C17FE84E41

*Opisotretus butteli* Carl, 1922 - Carl (1922): 573–574, figs. L–M.
*Solaenaulus butteli* (Carl, 1922) - Attems (1940): 172–173, fig. 246. Combination proposed by Attems (1940).
*Solaenaulus butteli birmanicus* Carl, 1941 - Carl (1941): 374–376, figs. 26–27.

#### Materials

- **Type status:** Other material. **Occurrence:** catalogNumber: KPM-NKB 2097; recordedBy: Yu Hisasue; individualCount: 1; sex: male; lifeStage: adult; occurrenceID: BEC72121-D5DB-5B8B-A234-5C007E0A8426; **Taxon:** scientificName: Solaenaulus butteli (Carl, 1922); kingdom: Animalia; phylum: Arthropoda; class: Diplopoda; order: Polydesmida; family: Opisotretidae; genus: Solaenaulus; specificEpithet: butteli; **Location:** islandGroup: Ogasawara Islands; island: Haha-jima Island; country: Japan; countryCode: JP; stateProvince: Tokyo; county: Ogasawara; locality: Mt. Chibusa-yama; verbatimLocality: Mt. Chibusa-yama, Haha-jima Island, Ogasawara Islands, Tokyo, Japan; decimalLatitude: 26.653912; decimalLongitude: 142.160484; geodeticDatum: WGS84; georeferenceVerificationStatus: verified by collector; **Identification:** identifiedBy: Soma Chiyoda; dateIdentified: 2025–2026; **Event:** samplingProtocol: sifting; eventDate: 2024-06-12; year: 2024; month: 6; day: 12; **Record Level:** institutionCode: KPM; basisOfRecord: PreservedSpecimen
- **Type status:** Other material. **Occurrence:** catalogNumber: KPM-NKB 2105; recordedBy: Yu Hisasue; individualCount: 1; sex: male; lifeStage: adult; occurrenceID: 8AA0489E-8200-5D6D-93C9-0225AE483D90; **Taxon:** scientificName: Solaenaulus butteli (Carl, 1922); kingdom: Animalia; phylum: Arthropoda; class: Diplopoda; order: Polydesmida; family: Opisotretidae; genus: Solaenaulus; specificEpithet: butteli; **Location:** islandGroup: Ogasawara Islands; island: Chichi-jima Island; country: Japan; countryCode: JP; stateProvince: Tokyo; county: Ogasawara; locality: Tatsumizaki; verbatimLocality: Tatsumizaki, Chichi-jima Island, Ogasawara Islands, Tokyo, Japan; decimalLatitude: 27.039; decimalLongitude: 142.2219; geodeticDatum: WGS84; georeferenceVerificationStatus: verified by collector; **Identification:** identifiedBy: Soma Chiyoda; dateIdentified: 2025–2026; **Event:** samplingProtocol: collected from leaf litter; eventDate: 2025-09-28; year: 2025; month: 9; day: 28; **Record Level:** institutionCode: KPM; basisOfRecord: PreservedSpecimen

#### Diagnosis

**Figure 6.**
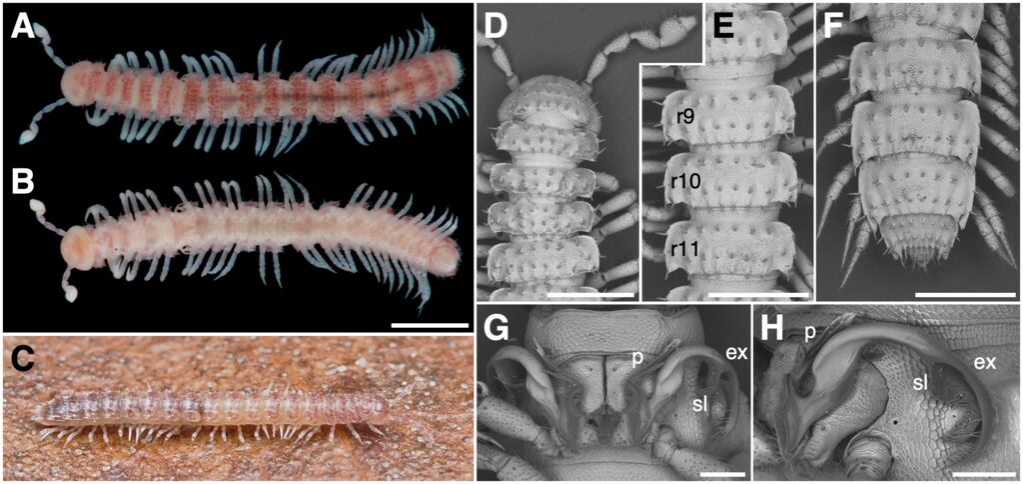
*Solaenaulus butteli* (Carl, 1922) from Ani-jima (**C**), Chichi-jima (**A, B, D–F**: KPM-NKB 2105) and Haha-jima Islands (**G, H**: KPM-NKB 2097). **A, B** Dorsal view (**A**) and ventral view (**B**) of male adult; **C** Female adult in life, photographed in Mt. Tachigami-yama, Ani-jima Island; **D–F** Dorsal view of anterior part (**D**), rings 9–11 (**E**) and posterior part (**F**); **G** Ventral view of intact gonopods; **H** Ventrolateral view of intact left gonopod. Abbreviations: ex, exomere; p, prefemoral process; r, ring (e.g. r9 = ring 9); sl, solenomere. Scale bars: 1 mm (**A, B**); 500 μm (**D–F**); 100 μm (**G, H**).

Based on the descriptions by Carl (1922), Carl (1941), Golovatch et al. (2010) and Golovatch et al. (2013), the specimens were identified as *S. butteli* by the following characters: males with head + 19 rings, 4.8–6.0 mm long (Fig. 6A and B); female with head + 20 rings, 6.2–6.7 mm long (Fig. 6C); colour smoky pink (Fig. 6A and C); shoulders of paranota usually not so prominent (Fig. 6A and D–F); gonopod telopodite bipartite (exomere and solenomere), with a basal process on frontoventral face of femorite prominent (Fig. 6G and H); the basal process relatively short, less than one-third of total length of telopodite (Fig. 6G and H).

#### Distribution

Myanmar, Singapore, Indonesia (Sumatra; type locality: “Säntis (Distrikt Deli). Ostsumatra”), Christmas Island (Indian Ocean), Papua New Guinea, Fiji, Japan (Ototo-jima Island, Ani-jima Island, Chichi-jima Island, Haha-jima Island) (Carl 1922, Carl 1941, Takano 1988, Jeekel 2006, Golovatch et al. 2010, Akkari and Enghoff 2011, Decker 2013, present study) (Fig. 1).

#### Ecology

This species was collected from beneath fallen logs and within leaf litter in forested areas. The collection sites were located relatively distant from settlements and the port.

#### Taxon discussion

Carl (1941) described *S. butteli birmanicus* from Myanmar as a subspecies of *S. butteli*, based on differences in the degree of development of the teeth on the paranota and in the morphology of the gonopods. Jeekel (2006) regarded the gonopodal differences between the two taxa as merely due to differences in the angle of illustration and, thus, did not recognise the subspecies, treating only *S. butteli* as valid. In contrast, Golovatch et al. (2010) treated *S. b. birmanicus* as a distinct species and recognised two species within the genus, *S. butteli* and *S. birmanicus*. As the gonopods illustrated by Golovatch et al. (2010) from different angles appeared to differ markedly in shape and because they reported that the New Guinean specimens possessed a combination of characters of both *S. butteli* and *S. birmanicus*, we follow Jeekel (2006) in recognising only *S. butteli* as valid within this genus. The specimens from the Ogasawara Islands reported by Takano (1988) and the present study both closely matched the gonopod morphology depicted in the original description of *S. butteli* rather than that of *S. b. birmanicus*, illustrated by Carl (1941).

#### Notes

##### Japanese name

We herein propose the new Japanese name “Yari-himeobiyasude” (Japanese: ヤリヒメオビヤスデ) for this species. This Japanese name refers to the characteristic basal process of the gonopod observed in this species. Furthermore, the genus *Solaenaulus* was given the Japanese name “Ogasawara-chibiyasude-zoku” (Japanese: オガサワラチビヤスデ属) by Takano (1988); however, because this species and the genus are not restricted to the Ogasawara Islands, but are widely distributed throughout Southeast Asia, we propose to revise the Japanese name of the genus to “Yari-himeobiyasude-zoku” (Japanese: ヤリヒメオビヤスデ属).

##### Reassessment of the record by Takano (1988)

Takano (1988) recorded *Solaenaulus* sp. from Chichi-jima Island in the Ogasawara Islands and noted its resemblance to *S. butteli*, while suggesting that it might represent an undescribed species because of the presence of a spine-like projection on the basal process of the gonopod. However, since the original description of *S. butteli* clearly depicts a spine-like projection and no distinguishing differences were found between the specimens from Chichi-jima Island and *S. butteli* (Carl 1922, Takano 1988), the record is herein identified as *S. butteli*.

## Discussion

### Insights into the composition and formation of the millipede fauna of the Ogasawara Islands

The present study revealed the presence of five species of polydesmidan millipedes, *Prosopodesmus jacobsoni*, *P. panporus*, *Cryptocorypha ornata*, *Dyskolonius uniramus* and *Solaenaulus butteli*, in the Ogasawara Islands. These millipedes represent novel additions to the millipede fauna of the Ogasawara Islands and Japan. All five species were represented by multiple individuals, including juveniles, strongly suggesting that they are reproducing and have established populations in the Ogasawara Islands. However, their distributions within the islands differed markedly amongst species. *C. ornata* and *S. butteli* were recorded from all four surveyed islands, indicating widespread establishment, whereas *P. jacobsoni* was restricted to Chichi-jima Island, *P. panporus* to Haha-jima Island and *D. uniramus* to Ototo-jima and Haha-jima Islands. These contrasting distribution patterns may reflect differences in introduction history, dispersal ability or ecological requirements amongst species.

Amongst the five species treated here, *P. jacobsoni*, *C. ornata* and *S. butteli* are widely distributed in tropical regions of Southeast Asia and the Pacific. Of these, *P. jacobsoni* and *C. ornata* have previously been suggested to have expanded their distributions in association with human activities (Hoffman 1999, Andrew et al. 2024) and some populations of *S. butteli* have also been considered to be introduced (Golovatch et al. 2013). Therefore, the occurrence of these species in the Ogasawara Islands is most likely attributable to unintentional introduction associated with planting activities or soil transport. *Cryptocorypha ornata* was collected from Chichi-jima and Haha-jima Islands in 1990 and *S. butteli* was collected from Chichi-jima Island in 1977 (Takano 1988, Tanabe 1991), suggesting that at least these two species had already invaded and become established in the Ogasawara Islands by that time, but were previously overlooked.

In contrast to the three species discussed above, the distribution patterns of *P. panporus* and *D. uniramus* differ markedly. *P. panporus* has previously been known only from a hothouse of the Royal Botanic Gardens, Kew, UK and from the Cape York Peninsula, Australia (Mesibov 2012), making Haha-jima Island the third known locality for the species. Mesibov (2012) suggested that the Cape York Peninsula represents the native range of *P. panporus*, whereas the record from the UK hothouse likely resulted from accidental introduction with plants. Given the high diversity of the genus *Prosopodesmus* in Australia, this interpretation appears plausible and the occurrence on Haha-jima Island may likewise be attributable to human-mediated introduction. Nevertheless, because the distribution pattern of *P. panporus* differs substantially from that of the three widely distributed species discussed above, it is possible that this species differs in its tolerance to disturbed environments and that its dispersal and establishment may be more limited.

*D. uniramus* was originally described from montane localities in Vietnam at elevations above 800 m (Attems 1938a) and no records have been reported since the original description. The present study therefore represents the first record of this species in 88 years. In the Ogasawara Islands, *D. uniramus* was collected from Ototo-jima Island, which is currently uninhabited and from relatively undisturbed forests on Haha-jima Island, but not from disturbed habitats, such as settlements or agricultural areas. These observations suggest that this species may have occurred naturally in the Ogasawara Islands; however, the possibility of introduction associated with human activities cannot be excluded and, at present, it is difficult to discuss how such a distribution pattern was established.

Based on the evidence currently available, only very limited inferences can be made regarding the processes underlying the establishment of these distribution patterns. To discuss the origins of these millipedes in greater depth, it would be necessary to apply molecular phylogenetic approaches, such as estimating genetic distances amongst populations from different localities and assessing the degree of genetic differentiation within the Ogasawara Islands.

With the addition of five species recorded in this study and the removal of the previous record of *Prosopodesmus sinuatus*, the number of millipede species recorded from the Ogasawara Islands now totals 14 (Table 1). Of these, only a single species, *Nedyopus boninensis*, is currently considered endemic to the Ogasawara Islands. Amongst the species previously recorded, *Asiomorpha coarctata* and *Pseudospirobolellus avernus* are both widely distributed in the tropical regions of Asia (Hoffman 1999) and *Oxidus gracilis* is cosmopolitan (Takakuwa 1942a, Hoffman 1999). Their distribution patterns strongly suggest that these species are of introduced origin. Thus, based on the evidence currently available, the millipede fauna of the Ogasawara Islands can be characterised by an extremely low level of endemism, with at least about half of the recorded species likely derived from human-mediated introductions. However, millipede surveys in the Ogasawara Islands have so far been very limited and the discovery of as many as five additional species during a short-term survey in this study strongly suggests that the total number of recorded species will increase further with continued investigation. Several unidentified species have already been noted (Tanabe 1991) and additional surveys will be essential to update our understanding of the characteristics of the fauna and the processes underlying its formation.

### Variation in ring number in Prosopodesmus spp.

In the present study, numerous juvenile specimens of *P. jacobsoni* and *P. panporus* were collected, allowing a comparison of their post-embryonic development with respect to adult ring number. As previously noted by Blower and Rundle (1980), adult males and females of *P. jacobsoni* possess 20 body rings, whereas adult males of *P. panporus* have only 19 rings, with females retaining 20 rings. As millipedes undergo anamorphosis, adding body rings at each moult, differences in adult ring number directly reflect differences in the timing of sexual maturation during postembryonic development. Our observations confirm that males of *P. jacobsoni* reach adulthood at stadium VIII, consistent with the typical pattern for Polydesmida (Enghoff et al. 1993), whereas males of *P. panporus* attain adulthood one stadium earlier, at stadium VII.

Within the genus *Prosopodesmus*, all species for which adult morphology has been documented, with the sole exception of *P. panporus*, possess males with 20 body rings (Miyosi 1958, Mesibov 2012). This distribution strongly suggests that 20 rings in adult males represents the ancestral condition in the genus and that the reduced ring number in *P. panporus* is a derived state. The most parsimonious explanation for this reduction is a heterochronic shift in the timing of sexual maturation, whereby males of *P. panporus* mature one stadium earlier than in congeners, such as *P. jacobsoni*. Comparable intrageneric variation in adult male ring number (19 vs. 20 rings) has been reported in other polydesmidan genera, including *Eutrichodesmus* and *Cryptocorypha* (Golovatch et al. 2009a, Golovatch 2019), suggesting that heterochronic evolution of this kind may have arisen repeatedly within the order.

Comparative analysis of gonopod development between *P. jacobsoni* and *P. panporus* provides additional support for this interpretation. When corresponding developmental stages are compared, gonopod primordia in *P. panporus* appear to be advanced by one stadium relative to those of *P. jacobsoni*. This shift in the developmental schedule of gonopod formation is consistent with the earlier attainment of sexual maturity in *P. panporus* and the concomitant reduction in adult ring number.

In polydesmidan millipedes that reach adulthood at stadium VIII, gonopod primordia typically appear at stadium IV and complete development by stadium VIII (Petit 1976, Drago et al. 2011). Referring to the stadial structure of *P. panporus* provided by Blower and Rundle (1980), gonopod primordia also appear to emerge at stadium IV in this species. This suggests that, in *P. panporus*, the onset of gonopod development is not shifted to an earlier stadium; rather, gonopod development is likely accelerated, resulting in completion by stadium VII. In species that attain adulthood at stadium VII, such as *Brachydesmus superus* (Polydesmidae), gonopod primordia likewise appear at stadium IV (Petit 1976). Such acceleration of sexual character development may represent a common mechanism underlying the repeated evolutionary shifts towards earlier sexual maturation observed in Polydesmida. As stadium IV and V juveniles of *P. panporus* were not available in the present study, further observations will be required to confirm the nature and extent of this developmental acceleration.

The present study provides the first detailed interspecific comparison of post-embryonic development associated with variation in adult ring number within the genus *Prosopodesmus*. The evolution of ring number has long been a central topic in myriapod biology and the presence of closely-related species differing in adult ring number makes *Prosopodesmus* a promising system for investigating the developmental and evolutionary mechanisms underlying this variation from an evolutionary developmental biology perspective.

## Supplementary Material

XML Treatment for Prosopodesmus jacobsoni

XML Treatment for Prosopodesmus panporus

XML Treatment for Cryptocorypha ornata

XML Treatment for Dyskolonius uniramus

XML Treatment for Solaenaulus butteli

1A56E615-B2F7-542A-8115-23C53E986FE0 10.3897/BDJ.14.e194219.suppl1 Supplementary material 1 Detailed list of examined specimens Data type occurrences Brief description Full list of all specimens examined in this study, including those specifically mentioned in the Materials section of the main text. File: oo_1655011.xls https://binary.pensoft.net/file/1655011 Soma Chiyoda, Yu Hisasue, Hiroki Yoshino, Ryosuke Kuwahara, Toru Miura

## Figures and Tables

**Table 1.** Millipedes recorded in the Ogasawara Islands, Japan. The species names listed in this table represent the currently accepted scientific names and do not necessarily correspond to those mentioned in the original literature. For records considered to be misidentifications, the names regarded as correct are given, with the rationale provided in the remarks column. Abbreviations for islands: A, Ani-jima Island; C, Chichi-jima Island; H, Haha-jima Island; M, Muko-jima Island; O, Ototo-jima Island. “Unknown” indicates that detailed specimen data were lacking; although the specimen was known to have been collected from the Ogasawara Islands, the specific island could not be determined.

Order	Family	Species (accepted name), morphotype	Literature	Recorded islands	Remarks
Polyxenida	Lophoproctidae	*Lophoturus okinawai* (Nguyen Duy-Jacquemin & Condé, 1982)	Ishii (1988)	C	
Polyxenida	Lophoproctidae	*Lophoturus okinawai* (Nguyen Duy-Jacquemin & Condé, 1982)	Ishii (1997)	C	Original data are same as Ishii (1988).
Polyzoniida	Siphonotidae	*Rhinotus okabei* (Takakuwa, 1942)	Takakuwa (1942b)	Unknown	Shinohara et al. (2015) suggested that this species may be a synonym of *R. purpureus*; however, due to insufficient justification, this treatment is not followed here.
Polyzoniida	Siphonotidae	*Rhinotus okabei* (Takakuwa, 1942)	Takakuwa (1943)	Unknown	
Polyzoniida	Siphonotidae	*Rhinotus okabei* (Takakuwa, 1942)	Tanabe (1991)	C	
Polydesmida	Xystodesmidae?	Considered close to Xystodesmidae	Tanabe (1991)	O	Only one female juvenile was collected.
Polydesmida	Haplodesmidae	*Prosopodesmus jacobsoni* Silvestri, 1910	Present study	C	
Polydesmida	Haplodesmidae	*Prosopodesmus panporus* Blower & Rundle, 1980	Present study	H	
Polydesmida	Polydesmidae	*Epanerchodus* sp.	Tanabe (1991)	A, C, H	Multiple individuals including one male were collected.
Polydesmida	Pyrgodesmidae	*Cryptocorypha ornata* (Attems, 1938)	Tanabe (1991)	C, H	Originally recorded as *Rhipidopeltis sinuata* (= *Prosopodesmus sinuatus*), but reexamination of the specimens identified them as *C. ornata* in the present study.
Polydesmida	Pyrgodesmidae	*Cryptocorypha ornata* (Attems, 1938)	Present study	O, A, C, H	
Polydesmida	Pyrgodesmidae	*Dyskolonius uniramus* (Attems, 1938)	Present study	O, H	
Polydesmida	Opisotretidae	*Solaenaulus butteli* (Carl, 1922)	Takano (1988)	C	Originally recorded as *Solaenaulus* sp., but identified as *S. butteli* based on detailed sketches in the present study.
Polydesmida	Opisotretidae	*Solaenaulus butteli* (Carl, 1922)	Present study	O, A, C, H	
Polydesmida	Paradoxosomatidae	*Helicorthomorpha holstii* (Pocock, 1895)	Takakuwa (1943)	Unknown	
Polydesmida	Paradoxosomatidae	*Helicorthomorpha holstii* (Pocock, 1895)	Takashima (1950)	H	Originally recorded as *Helicorthomorpha* sp., but Kuwahara and Hoshino (2025) and Kuwahara et al. (2025) regarded this record as *H. holstii*.
Polydesmida	Paradoxosomatidae	*Nedyopus boninensis* Verhoeff, 1940	Verhoeff (1940)	Unknown	
Polydesmida	Paradoxosomatidae	*Nedyopus boninensis* Verhoeff, 1940	Takakuwa (1943)	Unknown	
Polydesmida	Paradoxosomatidae	*Nedyopus boninensis* Verhoeff, 1940	Tanabe (1991)	C	
Polydesmida	Paradoxosomatidae	*Nedyopus boninensis* Verhoeff, 1940	Shinohara (1993)	Unknown	
Polydesmida	Paradoxosomatidae	*Asiomorpha coarctata* (de Saussure, 1860)	Takakuwa (1942a)	Unknown	
Polydesmida	Paradoxosomatidae	*Asiomorpha coarctata* (de Saussure, 1860)	Takakuwa (1943)	Unknown	
Polydesmida	Paradoxosomatidae	*Asiomorpha coarctata* (de Saussure, 1860)	Tanabe (1991)	M, H	
Polydesmida	Paradoxosomatidae	*Oxidus gracilis* (C. L. Koch, 1847)	Takakuwa (1943)	Unknown	
Julida	Julidae	*Anaulaciulus koreanus boninensis* (Verhoeff, 1939)	Verhoeff (1939)	Unknown	Although Paik (1958) suggested that the record may have resulted from a label mix-up, it is treated here as valid.
Julida	Julidae	*Anaulaciulus koreanus boninensis* (Verhoeff, 1939)	Takakuwa (1943)	Unknown	
Julida	Julidae	*Anaulaciulus* sp. 1	Tanabe (1991)	C	Only one female was collected.
Julida	Julidae	*Anaulaciulus* sp. 2	Tanabe (1991)	M	Only one female was collected.
Julida	Julidae	*Anaulaciulus* sp.	Nishimura and Takayama (2022)	C	Originally shown as Pseudospirobolellus cf. bulbiferus, but corrected as *Anaulaciulus* sp. based on the photograph.
Julida	Julidae	*Japanioiulus lobatus* Verhoeff, 1937	Takakuwa (1943)	Unknown	
Spirobolida	Pseudospirobolellidae	*Pseudospirobolellus avernus* (Butler, 1876)	Takakuwa (1940)	Unknown	
Spirobolida	Pseudospirobolellidae	*Pseudospirobolellus avernus* (Butler, 1876)	Takakuwa (1942a)	Unknown	
Spirobolida	Pseudospirobolellidae	*Pseudospirobolellus avernus* (Butler, 1876)	Takakuwa (1943)	Unknown	

**Table 2.** Stadial composition and changes in ring number and body length in *Prosopodesmus* spp. observed in the present study.

Species	Stadium	Number of collected individuals	Body ring formula	Number of leg pairs	Body length [mm]
*P. jacobsoni*	IV	3♂	15 (C+10+3+T)	♂16 / ♀-	♂2.2–2.3 / ♀-
	V	3♂2♀	17 (C+13+2+T)	♂22 / ♀23	♂2.7–2.9 / ♀2.7–3.0
	VI	10♂15♀	18 (C+15+1+T)	♂26 / ♀27	♂3.4–3.9 / ♀3.5–4.0
	VII	16♂16♀	19 (C+16+1+T)	♂28 / ♀29	♂4.2–4.8 / ♀4.3–4.9
	VIII	4♂6♀	20 (C+17+1+T)	♂30 / ♀31	♂5.3–5.7 / ♀5.4–5.8
*P. panporus*	VI	1♂	18 (C+15+1+T)	♂26 / ♀-	♂3.0 / ♀-
	VII	2♂	19 (C+16+1+T)	♂28 / ♀-	♂3.5–3.7 / ♀-
	VIII	1♀	20 (C+17+1+T)	♀31	♀3.5
